# Determinants of procedural sedation requirements during endovascular treatment for acute anterior circulation ischaemic stroke: a post-hoc analysis of the INDIVIDUATE trial

**DOI:** 10.1093/esj/aakag102

**Published:** 2026-08-31

**Authors:** Min Chen, David Batra, Nina Bieber, Lukas D Sauer, Arne Potreck, Markus Möhlenbruch, Peter Ringleb, Silvia Schönenberger

**Affiliations:** Department of Neurology, Heidelberg University Hospital, Heidelberg, Germany; Department of Neurology, Heidelberg University Hospital, Heidelberg, Germany; Department of Neurology, Heidelberg University Hospital, Heidelberg, Germany; Institute of Medical Biometry, Heidelberg University, Heidelberg, Germany; Department of Neuroradiology, Heidelberg University Hospital, Heidelberg, Germany; Department of Neuroradiology, Heidelberg University Hospital, Heidelberg, Germany; Department of Neurology, Heidelberg University Hospital, Heidelberg, Germany; Department of Neurology, Heidelberg University Hospital, Heidelberg, Germany

**Keywords:** analgosedation, acute ischaemic stroke, EVT

## Abstract

**Introduction:**

Procedural sedation during EVT for acute ischaemic stroke is widely used, but factors associated with analgosedative drug requirements remain poorly characterised. We aimed to identify patient and procedural factors associated with analgosedative drug requirements during EVT and explore their association with clinical outcomes.

**Patients and methods:**

We performed a post hoc analysis of the randomised INDIVIDUATE trial, including patients with anterior circulation ischaemic stroke undergoing EVT under procedural sedation. Sedation exposure was quantified using mean propofol, remifentanil and esketamine dose rates derived from protocolised 5-min medication records. Multivariable linear regression analyses were used to identify factors associated with sedation requirements. Sensitivity analyses were performed using body weight-normalised dose rates in patients with available body weight data. Exploratory analyses assessed associations between sedation requirements and procedural and clinical outcomes.

**Results:**

Of 250 patients enrolled in the INDIVIDUATE trial, 239 were included. In multivariable analyses, male sex was associated with higher propofol and esketamine dose rates; however, after body weight-normalisation, the association with propofol was no longer statistically significant. In weight-normalised sensitivity analyses including 198 patients (82.8%), male sex, ICA + M2 occlusion compared with M1 occlusion and pre-stroke disability remained associated with higher weight-normalised esketamine dose rates (β = 2.39 μg/kg/min, *P* = .021; β = 13.56 μg/kg/min, *P* < .001; and β = 2.82 μg/kg/min, *P* = .028, respectively). Additionally, the number of thrombectomy attempts was associated with higher remifentanil dose rates (β = 0.56 ng/kg/min, *P* = .010). Higher body weight-normalised esketamine dose rates were associated with less favourable NIHSS change at 24 h (β = 0.17, *P* = .022). Sedation requirements were not associated with 3-month functional outcome or mortality.

**Conclusion:**

Sedation requirements during EVT appeared to vary according to patient- and procedure-related factors, including sex, occlusion pattern, pre-stroke disability and procedural complexity. Sedation intensity was not associated with long-term clinical outcomes, supporting the use of adequate procedural sedation when clinically indicated.

**Clinical Trial Registration:**

Clinicaltrials.gov; NCT04578288.

## Graphical Abstract

**Graphical Abstract:**
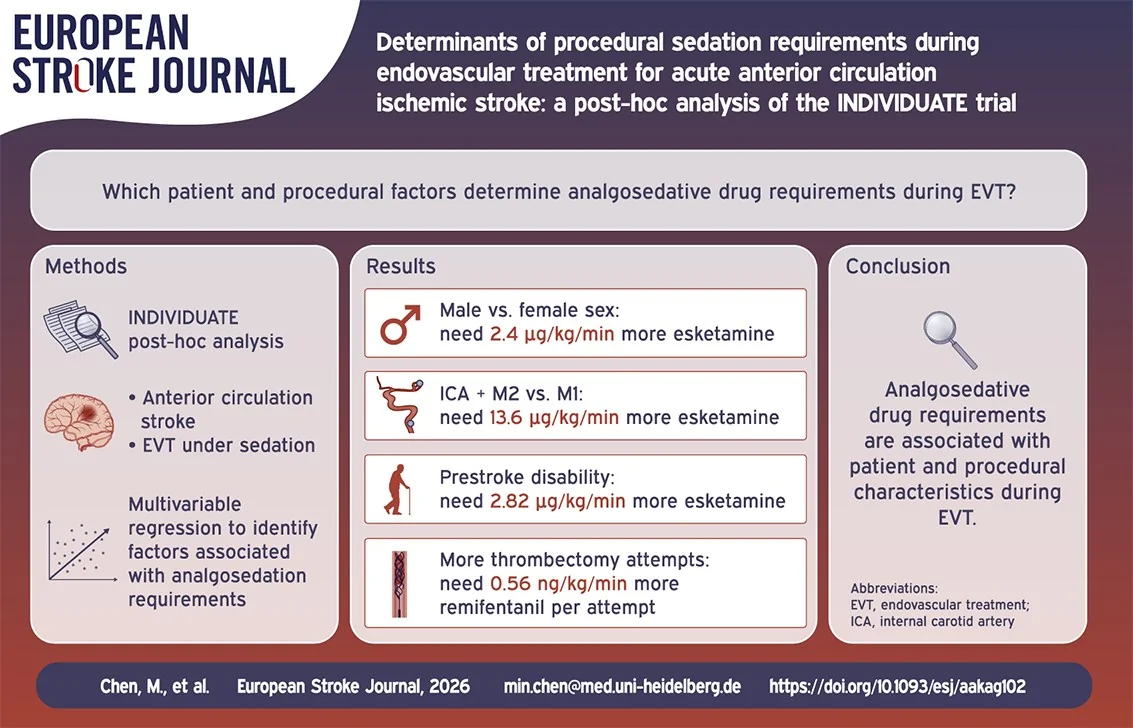

## Introduction

Optimal anaesthetic management for EVT remains a subject of ongoing debate.^1^ Although EVT can be performed under general anaesthesia, non-general anaesthesia approaches such as monitored anaesthesia care and moderate sedation represent established alternatives.^2^^,^^3^ Randomised controlled trials comparing these strategies have yielded conflicting results, leaving the optimal anaesthetic approach uncertain.^4–9^ Potential advantages of non-general anaesthesia include earlier neurological assessment, reduced need for intensive care resources and greater haemodynamic stability. Procedural sedation can be challenging, as insufficient sedation may disrupt procedural flow due to patient movement and lead to complications, whereas excessive sedation may require emergency intubation for airway protection and mechanical ventilation, which can be difficult in this acute setting.^10^^,^^11^

Anaesthetic strategies vary across centres, often reflecting historical practice patterns, institutional routines and available post-procedural care capacities.^2^^,^^12^^,^^13^ However, detailed descriptions of analgosedative drug regimens used during procedural sedation are scarce in the literature.

In this study, we aimed to analyse the characteristics of analgosedative drug use in patients enrolled in the INDIVIDUATE trial. This randomised controlled trial investigated blood pressure management strategies during EVT of anterior circulation vessel occlusion in a well-defined patient population, in which procedural sedation using a standardised institutional drug regimen constituted the standard approach.

## Methods

### Study design

Patients with acute ischaemic stroke of the anterior circulation with a NIHSS score of at least 8 and who underwent EVT were recruited between 1 October 2020 and 7 July 2022 at the Heidelberg University Hospital, Department of Neurology, Germany. Participants had to meet all of the following inclusion criteria: decision for thrombectomy according to local protocol for acute recanalising stroke treatment, age of 18 years or older, NIHSS ≥ 8, acute ischaemic stroke in the anterior circulation with isolated or combined occlusion of internal carotid artery (ICA) and/or middle cerebral artery. Informed consent from the patient or his/her legal representative was obtained within 72 h of treatment. Patients meeting the following criteria were excluded: ICH, coma on admission (Glasgow Coma Scale ≤ 8), severe respiratory instability, loss of airway protective reflexes or vomiting on admission for which primary intubation and general anaesthesia was deemed necessary and an intubated state before randomisation, as well as severe haemodynamic instability. Patients were randomised in a 1:1 ratio to either keeping the intraprocedural systolic blood pressure within the target range of a baseline SBP ± 10 mmHg (intervention) or to a standard blood pressure management (control group) where intraprocedural systolic blood pressure was to be maintained between 140 and 180 mmHg. During the recruitment period, 760 patients were screened for eligibility. Of these, 123 patients were randomised to individualised blood pressure management and 127 to standard blood pressure management. The primary endpoint 3-month mRS score of 0–2 was achieved in 31 patients in the individualised group and 30 patients in the control group, corresponding to a non-significant adjusted odds ratio of 0.81 (*P* = .56). Further details of the study have been published previously.^14^^,^^15^

Periprocedural care, including procedural sedation, was delivered by neurointensivists throughout the EVT procedure. The standard analgosedative regimen at our centre, which was also employed in the INDIVIDUATE trial, consisted of continuous propofol infusion with supplemental bolus administration as needed, combined with continuous remifentanil infusion. In cases of patient movement or insufficient sedation, additional propofol boluses were administered at the discretion of the treating neurointensivist. Esketamine served as a third-line adjunctive sedative and could be administered as a continuous infusion and/or intermittent bolus doses when adequate sedation could not be achieved with propofol and remifentanil alone.

Continuous infusion rates and bolus administrations were documented in the anaesthesia protocol at 5-min intervals. Multiple bolus administrations could occur within a single 5-min documentation period. Following EVT, patients were routinely transferred to the stroke unit. Admission to the neurological intensive care unit was reserved for patients requiring emergent conversion to general anaesthesia, those with critical cardiorespiratory conditions, or when dictated by bed availability and logistical considerations.

### Statistical analysis

Continuous variables are presented as mean (SD) or median (IQR/range), and categorical variables as counts (%). Patients who were intubated during the intervention were excluded, because their sedative management differed fundamentally from that of non-intubated patients. Intraprocedural sedation exposure was quantified at the patient level as mean propofol (mg/min), remifentanil (μg/min) and esketamine (mg/min) dose rates. For each agent, cumulative doses from continuous infusions and bolus administrations recorded in protocolised 5-min intervals were divided by the intraprocedural duration, defined as the time from groin puncture to final reperfusion assessment. Mean dose infusion rates were selected to reflect sedation intensity independent of procedure duration as cumulative sedative doses are inherently dependent on procedure length, which is itself associated with clinical outcome. Sensitivity analyses using weight-normalised dose rates were performed in patients with available body weight to account for the potential confounding effect of body weight, although body weight data were unavailable for 41 patients.

To identify factors associated with sedation dose rate, univariable linear regression analyses were performed separately for mean propofol, remifentanil and esketamine dose. Candidate exposures were age, sex, baseline NIHSS, pre-interventional ASPECTS, occlusion site, occlusion side, pre-stroke functional status dichotomised as pre-stroke mRS 0–2 vs 3–5, current smoking, pre-interventional CTA collateral score and number of thrombectomy attempts. Furthermore, multivariable linear regression analyses were performed. The primary multivariable models included the following prespecified baseline covariates only: age, sex, baseline NIHSS, pre-interventional ASPECTS, occlusion site and pre-stroke mRS (0–2 vs 3–5). Further exploratory multivariable models additionally included current smoking status, number of thrombectomy attempts and occlusion side. Additional univariable and multivariable models were performed using the NIHSS item 1a (Level of Consciousness/Vigilance) score as the determinant of vigilance.

Further exploratory outcome analyses were then performed using mean propofol, remifentanil and esketamine dose as exposure variables. Associations with 3-month mRS were assessed using ordinal logistic regression, successful reperfusion status (modified treatment in cerebral ischaemia [mTICI] 2b–3) and favourable functional outcome (mRS 0–2 vs 3–6) and death by 3 months using binary logistic regression. Associations with NIHSS at 24 h and change in NIHSS at 24 h were analysed using linear regression. Results are reported as beta coefficients or odds ratios with 95% CIs and 2-sided *P*-values. All models were fit on the set of complete cases without imputation of missing values. As the number of missing values was small, the putative resulting bias was considered negligible. Statistical significance was defined as a 2-sided *P*-value < .05. A correction for multiple testing was not applied, as all analyses were considered exploratory. As the primary objective of the study was to identify factors associated with sedation requirements, analyses examining associations between sedation requirements and clinical outcomes were performed for exploratory purposes only and were not intended to estimate causal effects of sedation intensity on outcome. Given the uncertainty regarding an appropriate adjustment set and the potential for overadjustment of variables related to procedural complexity and treatment success, outcome analyses were restricted to unadjusted models and should be considered hypothesis-generating. All analyses were performed in R (version 4.4.1).^16^

## Results

### Patient characteristics

Of the 250 patients in the INDIVIDUATE trial, 10 were excluded because they were intubated during the intervention, and 1 additional patient was excluded because of incomplete protocol documentation, so 239 patients were analysed.

Mean (SD) age of the study population was 77.4 (12.2) years, 136 (56.9%) were female and mean (SD) NIHSS at baseline was 16.6 (5.2). A total of 174 patients (72.8%) had no pre-stroke disability, defined as a pre-stroke mRS score of 0–2. Successful reperfusion was achieved in 210 (87.9%) patients and 57 (23.8%) had an mRS score of 0–2 after 3 months. Further demographical and clinical characteristics are described in Table 1. Body weight was available in 198 patients; their characteristics are shown in Table S1.

**Table 1:** Patient characteristics.

Patient characteristics	All patients *n* = 239
**Age, years, mean (SD)**	77.4 (12.2)
**Sex, female, n(%)**	136 (56.9%)
**Weight, female, kg, mean (SD)^a^**	69.9 (16.0)
**Weight, male, kg, mean (SD)^a^**	83.5 (18.2)
**Pre-stroke mRS 0–2, *n* (%)**	174 (72.8%)
**Baseline NIHSS, mean (SD)**	16.6 (5.2)
**Pre-interventional ASPECTS, mean (SD)**	8.3 (1.6)
**Pre-interventional CTA-CS, mean (SD)^b^**	1.8 (1.0)
**Medical history, *n* (%)**	
**Hypertension**	179 (74.9)
**Diabetes**	55 (23.0)
**Hypercholesterolaemia**	70 (29.3)
**Previous stroke or TIA**	44 (18.4)
**Atrial fibrillation^c^**	104 (43.7)
**Current smoking**	33 (13.8)
**Occlusion site, *n* (%)**	
**Internal carotid artery**	18 (7.5)
**M1 segment**	98 (41.0)
**M2 segment**	58 (24.3)
**Carotid T**	39 (16.3)
**Internal carotid artery and M1 or M2 segment**	25 (10.5)
**Other**	1 (0.4)
**Left-sided occlusion, *n* (%)**	149 (62.3)
**Number of thrombectomy attempts, mean (SD)^d^**	2.6 (2.5)
**Intravenous thrombolysis, *n* (%)**	106 (44.4)
**Time metrics, mean (SD), min**	
**Onset/last seen well to admission^e^**	395.4 (427.8)
**Admission to groin puncture^f^**	72.8 (133.8)
**Groin puncture to final reperfusion**	81.7 (57.2)
**Outcome parameters**	
**Successful reperfusion (mTICI 2b–3), *n* (%)**	210 (87.9)
**3-month mRS 0–2, *n* (%)**	57 (23.8)
**3-month mortality, *n* (%)**	71 (29.7)
**NIHSS at 24 h, mean (SD)^g^**	12.2 (7.7)

Abbreviations: ASPECTS = Alberta Stroke Program Early CT Score; CTA-CS: CTA Collateral Score; ICA = internal carotid artery; mTICI = modified treatment in cerebral ischaemia.

^a^Forty-one missing.

^b^Five missing.

^c^One missing.

^d^One missing.

^e^Two missing.

^f^Five missing.

^g^Seven missing

### Characteristics of analgosedative drug application

Most patients (*n* = 140 [58.6%]) received procedural sedation with propofol in combination with remifentanil. Seventy-one (29.7%) were sedated with propofol, remifentanil and esketamine. Three (1.3%) patients were not sedated (see Table 2). The median (IQR) number of 5-min intervals with a bolus application was 2 (1–4) for propofol and 1 (1–1) for esketamine. A median (IQR) bolus dose of 20 (10–20) mg propofol and 25 (25–25) mg esketamine was needed in a 5-min interval in patients receiving sedation bolus. Mean (SD) propofol dose rate per patient was 2.43 (1.4) mg/min during thrombectomy. Mean (SD) remifentanil dose rate was 1.12 (0.47) μg/min and esketamine dose rate was 1.00 (0.61) mg/min. Detailed sedation characteristics are depicted in Table 2 and Table S2.

**Table 2:** Sedation characteristics.

Intraprocedural sedation pattern	*n* (%) All patients *n* = 239
**Propofol, remifentanil and esketamine**	71 (29.7)
**Propofol and remifentanil only**	140 (58.6)
**Propofol and esketamine only**	0 (0.0)
**Remifentanil and esketamine only**	0 (0.0)
**Propofol only**	6 (2.5)
**Remifentanil only**	19 (7.9)
**Esketamine only**	0 (0.0)
**No propofol, remifentanil or esketamine**	3 (1.3)
**Patients with any propofol exposure**	217 (90.8)
**Patients with at least one propofol bolus entry**	170 (71.1)
**Patients with any esketamine exposure**	71 (29.7)
**Patients with at least one esketamine bolus entry**	23 (9.6)
**Intraprocedural dose rates**	
**Mean propofol dose rate among recipients, mg/min, mean (SD)**	2.43 (1.40)
**Mean weight-adjusted propofol dose rate among recipients, μg/kg/min, mean (SD)** ^**a**^	14.87 (6.31)
**Mean remifentanil dose rate among recipients, μg/min, mean (SD)**	1.12 (0.47)
**Mean weight-adjusted remifentanil dose rate among recipients, ng/kg/min, mean (SD)** ^**a**^	14.87 (6.31)
**Mean esketamine dose rate among recipients, mg/min, mean (SD)**	1.00 (0.61)
**Mean weight-adjusted esketamine dose rate among recipients, μg/kg/min, mean (SD)** ^**a**^	12.99 (8.10)

^a^Weight-normalised dose rate is available in *n* = 198 patients.

To explore whether higher sedative exposure was associated with haemodynamic conditions that could potentially affect cerebral perfusion or collateral filling, we added a descriptive analysis of intraprocedural systolic blood pressure, diastolic blood pressure, mean arterial pressure and heart rate among recipients of each sedative. Patients were stratified into lower and higher dose-rate groups using the median patient-level mean dose rate of the respective sedative as the cutoff (Table S3). Overall, haemodynamic parameters were similar across lower and higher dose-rate strata for each sedative.

### Association between patient factors and absolute and weight-normalised dose rates

In univariable analyses, age (β = −0.02 mg/min, *P* = .03), male sex (β = 0.60 mg/min, *P* = .002) and smoking status (β = 0.74 mg/min, *P* = .008) were significantly associated with mean propofol dose rate. Male sex (β = 0.14 μg/min, *P* = .033), pre-stroke disability (β = −0.17 μg/min, *P* = .019) and number of thrombectomy attempts (β = 0.04 μg/min, *P* = .003) were significantly associated with mean remifentanil dose rate. Male sex (β = 0.22 mg/min, *P* = .003) and tandem occlusion constellation of ICA + M2 vs M1 (β = 1.02 mg/min, *P* ≤ .001) were significantly associated with mean esketamine dose rate. Other exposure variables are shown in Table 3.

**Table 3:** Univariable regression analyses for different exposure factors with absolute dose rates.

Exposures	*N*	Propofol (mg/min)		Remifentanil (μg/min)		Esketamine (mg/min)	
		Beta (95% CI)	*P*-value	Beta (95% CI)	*P*-value	Beta (95% CI)	*P*-value
**Age, years**	239	−0.02 (−0.03 to −0.00)	.030	−0.00 (−0.01 to 0.00)	.099	−0.00 (−0.01 to 0.00)	.413
**Male sex**	239	0.60 (0.22 to 0.97)	.002	0.14 (0.01 to 0.27)	.033	0.22 (0.08 to 0.36)	.003
**Baseline NIHSS**	239	0.01 (−0.02 to 0.05)	.478	0.00 (−0.01 to 0.02)	.621	−0.00 (−0.02 to 0.01)	.661
**Pre-interventional ASPECTS**	239	−0.10 (−0.22 to 0.02)	.101	−0.04 (−0.08 to 0.01)	.090	−0.02 (−0.06 to 0.03)	.487
**Occlusion site: ICA vs M1**	239	−0.37 (−1.13 to 0.39)	.342	0.02 (−0.24 to 0.27)	.901	−0.03 (−0.30 to 0.25)	.859
**Occlusion site: ICA + M1 vs M1**	239	0.63 (−0.09 to 1.36)	.088	−0.06 (−0.31 to 0.18)	.618	0.06 (−0.20 to 0.32)	.655
**Occlusion site: ICAg + M2 vs M1**	239	−0.28 (−1.64 to 1.07)	.683	0.25 (−0.21 to 0.71)	.291	1.02 (0.53 to 1.52)	<.001
**Occlusion site: carotid-T vs M1**	239	−0.19 (−0.75 to 0.37)	.508	0.07 (−0.12 to 0.26)	.495	0.16 (−0.05 to 0.36)	.137
**Occlusion site: M2 vs M1**	239	−0.15 (−0.64 to 0.34)	.559	−0.14 (−0.30 to 0.03)	.107	0.08 (−0.10 to 0.26)	.388
**Occlusion site: other vs M1**	239	−1.26 (−4.23 to 1.71)	.407	−0.59 (−1.59 to 0.42)	.255	−0.23 (−1.31 to 0.85)	.679
**Right-sided occlusion**	239	−0.19 (−0.59 to 0.20)	.338	0.02 (−0.11 to 0.16)	.726	−0.01 (−0.16 to 0.14)	.880
**Pre-stroke mRS 3–5 vs 0–2**	239	−0.12 (−0.55 to 0.31)	.592	−0.17 (−0.32 to −0.03)	.019	0.07 (−0.09 to 0.23)	.405
**Current smoker**	239	0.74 (0.19 to 1.29)	.008	0.17 (−0.02 to 0.35)	.083	0.13 (−0.08 to 0.33)	.237
**Pre-interventional CTA collateral score**	234	−0.15 (−0.36 to 0.05)	.134	−0.03 (−0.09 to 0.04)	.469	−0.04 (−0.12 to 0.03)	.244
**Number of thrombectomy attempts**	238	0.01 (−0.07 to 0.09)	.806	0.04 (0.01 to 0.07)	.003	0.01 (−0.01 to 0.04)	.325

Abbreviations: ASPECTS = Alberta Stroke Program Early CT Score; ICA = internal carotid artery.

In the primary multivariable analysis (see Table 4) of propofol dose rate with predefined exposure factors such as age, sex, baseline NIHSS, pre-interventional ASPECTS, occlusion sites and pre-stroke functional dependency (mRS score 3–5), only male sex showed a significant association (β = 0.59 mg/min, *P* = .005). No significant associations could be found in the primary multivariable model for remifentanil dose rate. Male sex (β = 0.22 mg/min, *P* = .005) and ICA + M2 vs M1 (β = 0.92 mg/min, *P* < .001) were significantly associated with esketamine dose rate in the primary multivariable model.

**Table 4:** Primary multivariable regression analysis with age, male sex, baseline NIHSS, pre-interventional ASPECTS, occlusion site, pre-stroke functional status as covariables and absolute dose rates and weight-normalised dose rates as outcome. Analyses were performed with *n* = 239 cases for absolute dose rates and *n* = 198 for weight-normalised dose rates.

Exposures	Propofol (mg/min)		Remifentanil (μg/min)		Esketamine (mg/min)		Propofol (μg/kg/min)		Remifentanil (ng/hg/min)		Esketamine (μg/kg/min)	
	Beta (95% CI)	*P*-value	Beta (95% CI)	*P*-value	Beta (95% CI)	*P*-value	Beta (95% CI)	*P*-value	Beta (95% CI)	*P*-value	Beta (95% CI)	*P*-value
**Age, years**	−0.01 (−0.03 to 0.00)	.140	−0.00 (−0.01 to 0.00)	.515	−0.00 (−0.01 to 0.01)	.958	0.020 (−0.267 to 0.307)	.892	0.048 (−0.043 to 0.140)	.296	−0.006 (−0.095 to 0.083)	.896
**Male sex**	0.59 (0.18 to 0.99)	.005	0.11 (−0.03 to 0.25)	.108	0.22 (0.07 to 0.37)	.005	2.353 (−4.206 to 8.911)	.480	−1.205 (−3.289 to 0.879)	.256	2.392 (0.359 to 4.426)	.021
**Baseline NIHSS**	0.00 (−0.04 to 0.04)	.951	−0.00 (−0.01 to 0.01)	.920	−0.01 (−0.02 to 0.01)	.427	0.079 (−0.550 to 0.708)	.805	0.030 (−0.170 to 0.230)	.765	−0.046 (−0.241 to 0.150)	.646
**Pre-interventional ASPECTS**	−0.05 (−0.18 to 0.08)	.476	−0.03 (−0.07 to 0.02)	.212	−0.01 (−0.06 to 0.03)	.557	0.001 (−2.405 to 2.406)	1.000	0.034 (−0.731 to 0.798)	.931	−0.684 (−1.430 to 0.062)	.072
**Occlusion site: ICA vs M1**	−0.46 (−1.24 to 0.31)	.239	0.00 (−0.26 to 0.27)	.984	−0.04 (−0.32 to 0.25)	.787	−2.977 (−15.926 to 9.971)	.651	0.115 (−4.000 to 4.230)	.956	0.090 (−3.925 to 4.104)	.965
**Occlusion site: ICA + M1 vs M1**	0.38 (−0.37 to 1.13)	.320	−0.16 (−0.41 to 0.10)	.227	0.05 (−0.23 to 0.32)	.740	7.939 (−3.651 to 19.528)	.178	−0.950 (−4.633 to 2.733)	.611	0.655 (−2.938 to 4.248)	.720
**Occlusion site: ICA + M2 vs M1**	−0.82 (−2.22 to 0.59)	.253	0.12 (−0.36 to 0.60)	.619	0.92 (0.41 to 1.44)	<.001	−5.203 (−26.146 to 15.740)	.625	4.128 (−2.528 to 10.784)	.223	13.555 (7.062 to 20.049)	<.001
**Occlusion site: carotid−T vs M1**	−0.19 (−0.74 to 0.37)	.513	0.05 (−0.14 to 0.24)	.631	0.17 (−0.03 to 0.38)	.098	−2.374 (−11.571 to 6.823)	.611	−0.686 (−3.609 to 2.237)	.644	1.795 (−1.056 to 4.647)	.216
**Occlusion site: M2 vs M1**	−0.19 (−0.70 to 0.31)	.453	−0.13 (−0.30 to 0.05)	.149	0.05 (−0.14 to 0.24)	.603	−0.048 (−8.350 to 8.254)	.991	−0.633 (−3.271 to 2.006)	.637	1.997 (−0.578 to 4.571)	.128
**Pre-stroke mRS 3–5 vs 0–2**	0.16 (−0.32 to 0.65)	.505	−0.12 (−0.28 to 0.05)	.157	0.16 (−0.02 to 0.33)	.085	1.824 (−6.247 to 9.895)	.656	−2.103 (−4.668 to 0.462)	.107	2.818 (0.316 to 5.321)	.028

Abbreviations: ASPECTS = Alberta Stroke Program Early CT Score; ICA = internal carotid artery.

Further exploratory multivariable models with the addition of smoking status, number of thrombectomy attempts and occlusion side revealed no significant association of the added exposure factors, except a significant association of number of thrombectomy attempts and remifentanil rate (β = 0.03 μg/min, *P* = .019; weight-normalised β = 0.563 ng/kg/min, *P* = .010) (see Table S4).

In sensitivity analyses using body weight-normalised sedation dose rates, significant findings were largely consistent for remifentanil and esketamine. In univariable analyses, number of thrombectomy attempts was significantly associated with weight-normalised remifentanil dose rate (β = 0.54 ng/kg/min, *P* = .009), while male sex (β = 2.54 μg/kg/min, *P* = .013) and ICA + M2 vs M1 occlusion (β = 14.09 μg/kg/min, *P* < .001) were significantly associated with weight-normalised esketamine dose rate (see Table S5). In the primary multivariable weight-normalised models, male sex (β = 2.39 μg/kg/min, *P* = .021), ICA + M2 vs M1 occlusion (β = 13.56 μg/kg/min, *P* < .001) and pre-stroke disability (mRS 3–5 vs 0–2; β = 2.82 μg/kg/min, *P* = .028) were significantly associated with esketamine dose rate. In exploratory weight-normalised models, number of thrombectomy attempts remained significantly associated with remifentanil dose rate (β = 0.56 ng/kg/min, *P* = .010) and male sex (β = 2.46 μg/kg/min, *P* = .022), ICA + M2 vs M1 occlusion (β = 13.56 μg/kg/min, *P* < .001) and pre-stroke disability (β = 2.75 μg/kg/min, *P* = .034) remained significantly associated with esketamine dose rate. Exploratory analysis with vigilance showed no association with weight-normalised dose rates in univariable and multivariable analyses (see Table S6). Notably, none of the previously observed associations with propofol dose rate remained statistically significant after body weight-normalisation.

### Association between drug doses and clinical outcome

Long-term favourable functional outcome (mRS score 0−2) at 3 months and mortality by 3 months was not significantly associated with propofol, remifentanil or esketamine dose rates in univariable exploratory analysis (see Table 5).

**Table 5:** Explorative outcome models with absolute dose rate and weight-normalised dose rates as exposure. Change in NIHSS is NIHSS at 24 h − baseline NIHSS, positive values indicate unfavourable change. Analyses were performed with *n* = 239 cases except with *n* = 225 in the full cohort and *n* = 191 weight-normalised cohort cases in change in NIHSS at 24 h.

Outcome	Exposure	OR (95% CI)	*P*-value	Exposure	OR (95% CI)	*P*-value
**Favourable 3-month functional outcome (mRS 0–2 vs 3–6)**	Mean propofol dose rate, mg/min	1.11 (0.91 to 1.34)	.281	Mean weight-normalised propofol dose, μg/kg/min	1.00 (0.99 to 1.02)	.883
	Mean remifentanil dose rate, μg/min	0.93 (0.51 to 1.65)	.802	Mean weight-normalised remifentanil dose, ng/kg/min	0.98 (0.93 to 1.03)	.377
	Mean esketamine dose rate, mg/min	0.85 (0.47 to 1.45)	.580	Mean weight-normalised esketamine dose, μg/kg/min	0.98 (0.93 to 1.02)	.344
**3-month mortality**	Mean propofol dose rate, mg/min	0.90 (0.73 to 1.08)	.266	Mean weight-normalised propofol dose, μg/kg/min	1.00 (0.98 to 1.01)	.990
	Mean remifentanil dose rate, μg/min	1.07 (0.62 to 1.83)	.817	Mean weight-normalised remifentanil dose, ng/kg/min	1.01 (0.96 to 1.06)	.661
	Mean esketamine dose rate, mg/min	1.11 (0.67 to 1.78)	.669	Mean weight-normalised esketamine dose, μg/kg/min	1.02 (0.97 to 1.06)	.448
**Successful reperfusion (mTICI 2b–3)**	Mean propofol dose rate, mg/min	0.95 (0.75 to 1.24)	.708	Mean weight-normalised propofol dose, μg/kg/min	1.00 (0.97 to 1.02)	.711
	Mean remifentanil dose rate, μg/min	0.41 (0.20 to 0.85)	.016	Mean weight-normalised remifentanil dose, ng/kg/min	0.97 (0.90 to 1.04)	.344
	Mean esketamine dose rate, mg/min	0.84 (0.46 to 1.72)	.606	Mean weight-normalised esketamine dose, μg/kg/min	0.97 (0.92 to 1.04)	.354
		Beta (95% CI)			Beta (95% CI)	
**Change in NIHSS at 24 h**	Mean propofol dose rate, mg/min	−0.46 (−1.11 to 0.19)	.167	Mean weight-normalised propofol dose, μg/kg/min	−0.02 (−0.07 to 0.03)	.397
	Mean remifentanil dose rate, μg/min	1.48 (−0.46 to 3.42)	.135	Mean weight-normalised remifentanil dose, ng/kg/min	0.07 (−0.08 to 0.22)	.347
	Mean esketamine dose rate, mg/min	1.91 (0.19 to 3.63)	.030	Mean weight-normalised esketamine dose, μg/kg/min	0.17 (0.02 to 0.31)	.022

Abbreviations: mTICI = modified treatment in cerebral ischaemia; OR = odds ratio.

Higher remifentanil dose rate was associated with lower odds of successful reperfusion (OR 0.36; 95% CI, 0.16–0.77; *P* = .009), which did not remain significant in the weight-normalised model (*P* = .344). Higher esketamine dose rates were associated with less favourable change in NIHSS score at 24 h (β = 1.91 mg/min; 95% CI, 0.19–3.63; *P* = .03; weight-normalised β = 0.17 μg/kg/min; 95% CI, 0.02–0.31; *P* = .022). Further analyses are depicted in Table 5.

## Discussion

The present study identified several patient characteristics that may be associated with intraprocedural sedation requirements in patients undergoing endovascular therapy for anterior circulation stroke. In analyses of absolute dose rates, male sex was independently associated with higher propofol and esketamine dose rates after adjustment for multiple confounders. However, after body weight normalisation, the association between male sex and propofol dose rate was no longer statistically significant, suggesting that the observed association with absolute propofol dose may be partly explained by differences in body weight. In contrast, the association between male sex and weight-normalised esketamine dose rate persisted, suggesting higher overall esketamine requirements in male patients. This finding is intriguing, given previous evidence that anaesthetic responses may differ between sexes. A large meta-analysis^17^ reported a higher incidence of awareness under general anaesthesia and faster emergence from anaesthesia among women, while another study^18^ suggested that women may require higher propofol infusion rates to achieve comparable levels of sedation. Experimental evidence further indicates that anaesthetic sensitivity may be hormonally mediated.^19^ Taken together, the loss of significance for propofol after weight normalisation and the persistent association with weight-normalised esketamine dose rates indicate that the relationship between sex and sedative dose requirements may differ between sedatives. The higher esketamine requirements observed in male patients may reflect a more frequent need for escalation to a more complex sedation strategy during EVT, although this interpretation remains exploratory.

Another notable finding was that combined ICA + M2 tandem occlusions were associated with higher weight-normalised esketamine dose rates. One possible explanation is the increased procedural complexity of these cases, which often require carotid artery stenting in addition to intracranial thrombectomy.^20^ As these interventions are often longer and more technically demanding, achieving adequate procedural conditions may require greater sedation intensity, including the adjunctive use of esketamine in addition to propofol and remifentanil. However, this finding should be interpreted cautiously because the ICA + M2 subgroup was small (*n* = 5). Furthermore, higher remifentanil weight-normalised dose rates were associated with a greater number of thrombectomy attempts corresponding to an approximately 4% higher rate per additional attempt. This supports the interpretation that remifentanil requirements may also reflect procedural complexity.

The association between pre-stroke disability and higher weight-normalised esketamine dose rates is noteworthy. Patients with pre-stroke mRS score of 3–5 may represent a clinically more vulnerable subgroup with greater procedural complexity, reduced tolerance of discomfort or increased need for sedation escalation during EVT. Further studies are needed to determine whether pre-stroke functional dependency is independently associated with sedation escalation or whether this association reflects unmeasured clinical and procedural factors.

Interestingly, no association was observed between analgosedative drug requirements and the side of vessel occlusion. This finding is somewhat unexpected, as left-sided strokes are more commonly associated with aphasia, which could theoretically contribute to patient agitation by impairing the ability to understand instructions and the procedural context. One study reported that left-sided vessel occlusions were associated with a higher likelihood of undergoing EVT under general anaesthesia, an association that was largely mediated by aphasia. Based on these findings, the authors suggested that general anaesthesia may be preferentially considered in patients with left-sided occlusions, despite the less favourable discharge outcomes observed with general anaesthesia.^21^ In contrast, our data did not demonstrate an association between occlusion laterality and analgosedative drug requirements during procedural sedation. Although our findings cannot directly inform the choice between general anaesthesia and non-general anaesthesia strategies, they do not support the notion that left-sided occlusions inherently require greater sedation intensity. Consequently, occlusion laterality alone may be of limited value when selecting the anaesthetic approach for EVT.

No association was observed between analgosedative drug requirements and long-term clinical outcomes, including 3-month functional outcome and mortality. Although causality cannot be inferred, these findings provide some reassurance that the administration of higher analgosedative doses, when required to maintain adequate procedural conditions, does not appear to adversely affect patient outcomes.

In contrast, higher esketamine weight-normalised dose rates were associated with less favourable early neurological evolution at 24 h. These findings should be interpreted cautiously, given the exploratory and largely unadjusted nature of the analyses. Rather than reflecting independent adverse effects of esketamine, higher analgosedative requirements may represent surrogate markers of procedural complexity. More technically challenging and prolonged interventions are likely to require greater sedative and analgesic support while also being associated with less favourable early neurological outcomes. The absence of corresponding associations with long-term functional outcome or mortality suggests that these observations may primarily reflect short-term procedural factors rather than sustained adverse effects of the analgosedative agents themselves.

This study has several limitations. First, the analyses were conducted post hoc and were not prespecified in the original study protocol. Therefore, residual confounding and bias cannot be excluded. For example, the use of a third sedative agent (esketamine) may have reflected the anticipated complexity of the procedure, such as the need for carotid artery stenting in addition to intracranial thrombectomy, rather than true sedation requirements alone. Second, although the primary analyses quantified sedation exposure using absolute dose rates, body weight may represent an important confounder of sedative dose requirements. We therefore performed additional sensitivity analyses using weight-normalised dose rates in patients with available body weight data. However, these analyses should be interpreted cautiously, as body weight was not systematically collected and was unavailable in a substantial proportion of patients. Consequently, selection bias and residual confounding cannot be excluded.

In particular, the association between male sex and absolute propofol dose rate did not persist after body weight-normalisation, suggesting that this finding may partly reflect differences in body size. Conversely, the association between male sex and esketamine dose rate persisted after body weight-normalisation and warrants further investigation.

These limitations should be considered alongside several strengths. The study was conducted in a well-defined patient population undergoing EVT for anterior circulation stroke and employed a largely standardised procedural sedation strategy. In addition, sedative drug administration was documented at 5-min intervals, allowing for a relatively granular assessment of intraprocedural sedation exposure. The study was performed at a high-volume tertiary academic stroke centre, thereby reflecting routine clinical practice in a setting with substantial experience in procedural sedation during EVT. Furthermore, the inclusion of patients with pre-existing functional dependency increases the applicability of the findings to a broad spectrum of patients encountered in daily clinical practice.

## Conclusion

In this exploratory analysis of patients with anterior circulation stroke undergoing EVT under procedural sedation, higher sedation requirements were associated with patient- and procedure-related factors, including male sex, ICA + M2 tandem occlusion, pre-stroke disability and number of thrombectomy attempts. After body weight normalisation, the association between male sex and propofol dose rate was no longer significant, whereas male sex remained associated with higher esketamine dose rates. These findings warrant further investigation and may contribute to a better understanding of factors influencing sedation needs during EVT.

## Supplementary Material

Supplementary_material_aakag102

## Data Availability

Deidentified participant data can be obtained upon request from Silvia Schönenberger or Min Chen ending on 1 January 2030. Every investigator providing a methodologically sound proposal to test the data for confirmation or on a clinically relevant novel research question will be given access to the data repository after a positive evaluation of his/her request by Silvia Schönenberger and Min Chen. Proposals should be directed to either silvia.schoenenberger@med.uni-heidelberg.de or min.chen@med.uni-heidelberg.de. After approval, the requestor will have to sign a data access agreement. All documents are for a predetermined time, typically 12 months.
