# Supplementary material for: Determinants of procedural sedation requirements during endovascular treatment for acute anterior circulation ischaemic stroke: a post-hoc analysis of the INDIVIDUATE trial
Source: Eur Stroke J. 2026 Aug 31;11(8):aakag102. doi: 10.1093/esj/aakag102 (PMC13528861; doi:10.1093/esj/aakag102)
Supplement: Supplementary file 1 [file supplementary_material_aakag102.zip › INDIVIDUATE_Sedation_Supp_260803r1.docx]

Supplementary Material

For ‘Determinants of procedural sedation requirements during endovascular treatment for acute anterior circulation ischemic stroke. A post-hoc analysis of the INDIVIDUATE Trial’

Supplementary Table 1. Patient characteristics in the full study cohort and the sub cohort of patients with available weight data. ^a^ 5 missing ^b^ 1 missing ^c^ 1 missing ^d^ 1 missing ^e^ 2 missing ^f^ 6 missing ^g^ 5 missing ^h^ 14 missing ^i^ 7 missing

| **Patient characteristics** | All patients  N = 239 | Patients with weight data  N = 198 |
| --- | --- | --- |
| Age, years, mean (SD) | 77.4 (12.2) | 76.5 (12.5) |
| Sex, female, n(%) | 136 (56.9%) | 110 (55.6%) |
| Weight, female, kg, mean (SD) |  | 69.9 (16.0) |
| Weight, male, kg, mean (SD) |  | 83.5 (18.2) |
| Prestroke mRS 0-2, n(%) | 174 (72.8%) | 148 (74.7%) |
| Baseline NIHSS, mean (SD) | 16.6 (5.2) | 16.7 (5.2) |
| Preinterventional ASPECTS, mean (SD) | 8.3 (1.6) | 8.4 (1.4) |
| Preinterventional CTA-CS, mean (SD) | 1.8 (1.0) ^a^ | 1.8 (0.9) ^b^ |
| Medical history, n(%) |  |  |
| Hypertension | 179 (74.9) | 149 (75.3) |
| Diabetes | 55 (23.0) | 41 (20.7) |
| Hypercholesterolemia | 70 (29.3) | 54 (27.3) |
| Previous stroke or TIA | 44 (18.4) | 37 (18.7) |
| Atrial fibrillation^c^ | 104 (43.7) | 86 (43.7) |
| Current smoking | 33 (13.8) | 24 (12.1) |
| Occlusion site, n(%) |  |  |
| Internal carotid artery | 18 (7.5) | 14 (7.1) |
| M1 segment | 98 (41.0) | 81 (40.9) |
| M2 segment | 58 (24.3) | 49 (24.7) |
| Carotid T | 39 (16.3) | 30 (15.2) |
| Internal carotid artery and M1 or M2 segment | 25 (10.5) | 24 (12.1) |
| Other | 1 (0.4) | 0 (0) |
| Left-sided occlusion, n(%) | 149 (62.3) | 121 (61.1) |
| Number of thrombectomy attempts, mean (SD)^d^ | 2.6 (2.5) | 2.5 (2.4) |
| Intravenous thrombolysis, n(%) | 106 (44.4) | 104 (52.5) |
| Time metrics, mean (SD), min |  |  |
| Onset/last seen well to admission^e^ | 395.4 (427.8) | 348.3 (400.3) |
| Admission to groin puncture | 72.8 (133.8) ^f^ | 66.9 (104.9) ^g^ |
| Groin puncture to final reperfusion | 81.7 (57.2) | 77.2 (54.5) |
| Outcome parameters |  |  |
| Successful reperfusion (mTICI 2b-3), n(%) | 210 (87.9) | 183 (92.4) |
| 3-month mRS 0-2, n(%) | 57 (23.8) | 56 (28.3) |
| 3-month mortality, n(%) | 71 (29.7) | 48 (24.2) |
| NIHSS at 24 hours, mean (SD) | 12.2 (7.7) ^h^ | 11.4 (7.4) ^i^ |

Supplementary Table 2. Sedation characteristics in the full study cohort and the subcohort of patients with available weight data.

| **Intraprocedural sedation pattern** | **n (%)**  **All patients**  **n=239** | **n (%)**  **Patients with weight data**  **n=198** |
| --- | --- | --- |
| Propofol, remifentanil, and esketamine | 71 (29.7) | 54 (27.3%) |
| Propofol and remifentanil only | 140 (58.6) | 119 (60.1%) |
| Propofol and esketamine only | 0 (0.0) | 0 (0.0) |
| Remifentanil and esketamine only | 0 (0.0) | 0 (0.0) |
| Propofol only | 6 (2.5) | 5 (2.5%) |
| Remifentanil only | 19 (7.9) | 17 (8.6%) |
| Esketamine only | 0 (0.0) | 0 (0.0) |
| No propofol, remifentanil, or esketamine | 3 (1.3) | 3 (1.5%) |
| **Propofol characteristics** | |  |
| Patients with any propofol exposure | 217 (90.8) | 178 (89.9) |
| Patients with at least one propofol bolus entry | 170 (71.1) | 144 (72.7) |
| Number of 5-minute propofol bolus entries per patient receiving bolus, median [IQR] | 2 [1-4] | 2 [1-4] |
| Number of 5-minute propofol bolus entries per patient receiving bolus, range | 1-12 | 1-12 |
| Dose per 5-minute propofol bolus entry, mg, median [IQR] | 20 [10-20] | 20 [10-20] |
| Dose per 5-minute propofol bolus entry, mg, range | 4-120 | 4-120 |
| **Esketamine characteristics** | |  |
| Patients with any esketamine exposure | 71 (29.7) | 57 (27.3) |
| Patients with at least one esketamine bolus entry | 23 (9.6) | 17 (8.6) |
| Number of 5-minute esketamine bolus entries per patient receiving bolus, median [IQR] | 1 [1-1] | 1 [1-1] |
| Number of 5-minute esketamine bolus entries per patient receiving bolus, range | 1-6 | 1-4 |
| Dose per 5-minute esketamine bolus entry, mg, median [IQR] | 25 [25-25] | 25 [25-25] |
| Dose per 5-minute esketamine bolus entry, mg, range | 10-100 | 10-100 |
| **Intraprocedural sedation dose rates** | |  |
| Mean propofol dose, mg/min, mean (SD) | 2.21 (1.51) | 2.23 (1.55) |
| Mean propofol dose among recipients, mg/min, mean (SD) | 2.43 (1.40) | 2.48 (1.44) |
| Mean weight-adjusted propofol dose, µg/kg/min, mean (SD) |  | 30.68 (21.40) |
| Mean weight-adjusted propofol dose among recipients, µg/kg/min, mean (SD) |  | 34.13 (19.78) |
| Range of propofol dose among recipients, mg/min, | 0.19-9.11 | 0.22-9.11 |
| Mean remifentanil dose, µg/min, mean (SD) | 1.07 (0.51) | 1.05 (0.49) |
| Mean remifentanil dose among recipients, µg/min, mean (SD) | 1.12 (0.47) | 1.09 (0.45) |
| Mean weight-adjusted remifentanil dose, ng/kg/min, mean (SD) |  | 14.27 (6.84) |
| Mean weight-adjusted remifentanil dose among recipients, ng/kg/min, mean (SD) |  | 14.87 (6.31) |
| Range of remifentanil dose among recipients, µg/min | 0.08-3.13 | 0.08-3.13 |
| Mean esketamine dose, mg/min, mean (SD) | 0.30 (0.56) | 0.28 (0.55) |
| Mean weight-adjusted esketamine dose, µg/kg/min, mean (SD) |  | 3.54 (7.16) |
| Mean esketamine dose among recipients, mg/min, mean (SD) | 1.00 (0.61) | 1.03 (0.57) |
| Mean weight-adjusted esketamine dose among recipients, µg/kg/min, mean (SD) |  | 12.99 (8.10) |
| Range of esketamine dose among recipients, mg/min | 0.02-2.98 | 0.00-2.71 |

Supplementary Table 3. Intraprocedural hemodynamic parameters by lower versus higher sedative dose rate among recipients. For each sedative, only patients with any exposure to the respective drug were included. Within each sedative recipient group, patients were divided into lower and higher dose-rate strata using the median patient-level mean dose rate as the cutoff: propofol 2.12 mg/min, remifentanil 1.07 µg/min, and esketamine 0.93 mg/min. Patient-level dose rates were calculated over the complete duration of the intraprocedural phase.

| **Parameter** | **Propofol lower dose rate** | **Propofol higher dose rate** | **Remifentanil lower dose rate** | **Remifentanil higher dose rate** | **Esketamine lower dose rate** | **Esketamine higher dose rate** |
| --- | --- | --- | --- | --- | --- | --- |
| Sedative recipients in stratum | N = 109 | N = 108 | N = 115 | N = 115 | N = 36 | N = 35 |
| Median dose-rate cutoff | 2.12 | | 1.07 | | 0.93 | |
| Systolic BP, mean (SD), mmHg | 154.6 (21.1) | 156.1 (16.7) | 156.7 (19.4) | 154.6 (18.5) | 153.7 (14.3) | 157.8 (19.7) |
| Available systolic BP, n | 109 | 108 | 115 | 115 | 36 | 35 |
| Diastolic BP, mean (SD), mmHg | 84.9 (14.8) | 84.9 (12.1) | 84.7 (12.7) | 85.0 (14.3) | 87.2 (13.4) | 88.9 (12.7) |
| Available diastolic BP, n | 109 | 108 | 115 | 115 | 36 | 35 |
| Mean arterial pressure, mean (SD), mmHg | 108.1 (15.4) | 108.6 (12.2) | 108.7 (13.4) | 108.2 (14.3) | 109.3 (12.5) | 111.9 (13.4) |
| Available mean arterial pressure, n | 109 | 108 | 115 | 115 | 36 | 35 |
| Heart rate, mean (SD), bpm | 78.1 (17.8) | 77.3 (17.7) | 76.2 (17.6) | 77.7 (17.8) | 80.4 (19.1) | 78.9 (15.5) |
| Available heart rate, n | 109 | 106 | 115 | 113 | 36 | 35 |

Supplementary Table 4. Exploratory multivariable regression analysis with age, male sex, baseline NIHSS, preinterventional ASPECTS, occlusion site, prestroke functional status and additionally smoking status, number of thrombectomy attempts and occlusion side as covariables with absolute dose rates and weight-normalized dose rates of propofol, remifentanil and esketamine. Analyses were performed with n=239 complete cases in the cohort with absolute dose rates and n=191 with weight-normalized dose rates, except 1 missing in number of thrombectomy attempts.

| **Exposures** | **Propofol (mg/min)** | | **Remifentanil (µg/min)** | | **Esketamine (mg/min)** | | **Propofol (µg/kg/min)** | | **Remifentanil (ng/kg/min)** | | **Esketamine (µg/kg/min)** | |
| --- | --- | --- | --- | --- | --- | --- | --- | --- | --- | --- | --- | --- |
|  | **Beta (95% CI)** | **p value** | **Beta (95% CI)** | **p value** | **Beta (95% CI)** | **p value** | **Beta (95% CI)** | **p value** | **Beta (95% CI)** | **p value** | **Beta (95% CI)** | **p value** |
| Age, years | -0.01 (-0.03 to 0.01) | 0.253 | -0.00 (-0.01 to 0.00) | 0.624 | 0.00 (-0.01 to 0.01) | 0.538 | 0.064 (-0.233 to 0.361) | 0.669 | 0.062 (-0.031 to 0.155) | 0.188 | -0.001 (-0.093 to 0.092) | 0.991 |
| Male sex | 0.61 (0.14 to 1.08) | 0.011 | 0.11 (-0.02 to 0.24) | 0.093 | 0.33 (0.10 to 0.57) | 0.006 | 1.510 (-5.230 to 8.251) | 0.659 | -1.223 (-3.327 to 0.881) | 0.253 | 2.463 (0.367 to 4.559) | 0.022 |
| Baseline NIHSS | -0.02 (-0.07 to 0.02) | 0.278 | 0.00 (-0.01 to 0.01) | 0.838 | -0.01 (-0.03 to 0.02) | 0.511 | -0.004 (-0.667 to 0.659) | 0.990 | 0.059 (-0.148 to 0.266) | 0.575 | -0.048 (-0.254 to 0.159) | 0.650 |
| Preinterventional ASPECTS | -0.07 (-0.22 to 0.08) | 0.345 | -0.01 (-0.05 to 0.03) | 0.621 | 0.00 (-0.07 to 0.08) | 0.923 | -0.194 (-2.665 to 2.277) | 0.877 | 0.257 (-0.514 to 1.028) | 0.512 | -0.604 (-1.372 to 0.165) | 0.123 |
| Occlusion site: ICA vs M1 | -0.63 (-1.50 to 0.25) | 0.159 | -0.02 (-0.26 to 0.23) | 0.899 | -0.08 (-0.52 to 0.36) | 0.720 | -3.667 (-16.781 to 9.446) | 0.582 | 0.415 (-3.678 to 4.508) | 0.842 | 0.259 (-3.818 to 4.337) | 0.900 |
| Occlusion site: ICA+M1 vs M1 | 0.50 (-0.36 to 1.35) | 0.255 | -0.12 (-0.36 to 0.12) | 0.328 | 0.47 (0.04 to 0.91) | 0.032 | 6.994 (-4.893 to 18.882) | 0.247 | -1.047 (-4.757 to 2.664) | 0.579 | 0.781 (-2.915 to 4.477) | 0.677 |
| Occlusion site: ICA+M2 vs M1 | -1.10 (-2.68 to 0.48) | 0.171 | 0.10 (-0.34 to 0.54) | 0.658 | 1.04 (0.24 to 1.84) | 0.011 | -5.311 (-26.360 to 15.737) | 0.619 | 4.351 (-2.218 to 10.920) | 0.193 | 13.563 (7.018 to 20.107) | <0.001 |
| Occlusion site: Carotid-T vs M1 | -0.16 (-0.80 to 0.47) | 0.617 | 0.03 (-0.15 to 0.20) | 0.771 | 0.24 (-0.08 to 0.56) | 0.139 | -1.427 (-10.858 to 8.003) | 0.766 | -1.421 (-4.365 to 1.522) | 0.342 | 1.476 (-1.457 to 4.408) | 0.322 |
| Occlusion site: M2 vs M1 | -0.32 (-0.89 to 0.26) | 0.279 | -0.12 (-0.28 to 0.04) | 0.149 | 0.03 (-0.26 to 0.32) | 0.819 | -0.093 (-8.463 to 8.278) | 0.983 | -0.332 (-2.945 to 2.281) | 0.802 | 2.145 (-0.458 to 4.748) | 0.106 |
| Prestroke mRS 3-5 vs 0-2 | 0.21 (-0.34 to 0.75) | 0.458 | -0.11 (-0.26 to 0.04) | 0.156 | 0.37 (0.09 to 0.65) | 0.009 | 1.708 (-6.448 to 9.864) | 0.680 | -2.386 (-4.931 to 0.160) | 0.066 | 2.749 (0.213 to 5.285) | 0.034 |
| Current smoker | 0.25 (-0.44 to 0.94) | 0.471 | 0.09 (-0.10 to 0.29) | 0.342 | 0.26 (-0.09 to 0.61) | 0.147 | 7.116 (-3.215 to 17.446) | 0.176 | 1.396 (-1.828 to 4.620) | 0.394 | 0.190 (-3.022 to 3.402) | 0.907 |
| Number of thrombectomy attempts | 0.01 (-0.07 to 0.10) | 0.752 | 0.03 (0.01 to 0.06) | 0.012 | 0.02 (-0.02 to 0.07) | 0.286 | 0.008 (-1.359 to 1.375) | 0.991 | 0.563 (0.136 to 0.989) | 0.010 | 0.286 (-0.139 to 0.711) | 0.186 |
| Right-sided occlusion | -0.43 (-0.89 to 0.04) | 0.070 | 0.03 (-0.09 to 0.16) | 0.598 | -0.12 (-0.36 to 0.11) | 0.308 | -3.277 (-10.042 to 3.488) | 0.341 | 1.063 (-1.049 to 3.174) | 0.322 | 0.059 (-2.045 to 2.162) | 0.956 |

Supplementary Table 5. Univariable regression analyses for different exposure factors with weight-normalized mean dose rates.

| **Exposures** | **N** | **Propofol (µg/kg/min)** | | **Remifentanil (ng/kg/min)** | | **Esketamine (µg/kg/min)** | |
| --- | --- | --- | --- | --- | --- | --- | --- |
|  |  | **Beta (95% CI)** | **p value** | **Beta (95% CI)** | **p value** | **Beta (95% CI)** | **p value** |
| Age, years | 198 | -0.006 (-0.246 to 0.233) | 0.958 | 0.026 (-0.051 to 0.102) | 0.514 | -0.037 (-0.117 to 0.043) | 0.371 |
| Male sex | 198 | 1.968 (-4.040 to 7.976) | 0.522 | -0.777 (-2.697 to 1.142) | 0.428 | 2.542 (0.561 to 4.522) | 0.013 |
| Baseline NIHSS | 198 | 0.109 (-0.468 to 0.687) | 0.711 | 0.002 (-0.183 to 0.186) | 0.985 | -0.047 (-0.240 to 0.146) | 0.634 |
| Preinterventional ASPECTS | 198 | -0.512 (-2.693 to 1.669) | 0.646 | 0.130 (-0.567 to 0.827) | 0.715 | -0.534 (-1.260 to 0.192) | 0.151 |
| Occlusion site: ICA vs M1 | 198 | -2.621 (-14.815 to 9.573) | 0.674 | -0.306 (-4.212 to 3.600) | 0.878 | -0.211 (-4.110 to 3.687) | 0.915 |
| Occlusion site: ICA+M1 vs M1 | 198 | 7.650 (-3.089 to 18.389) | 0.164 | -1.419 (-4.859 to 2.021) | 0.420 | 1.239 (-2.194 to 4.672) | 0.480 |
| Occlusion site: ICA+M2 vs M1 | 198 | -4.959 (-24.373 to 14.454) | 0.617 | 2.905 (-3.314 to 9.124) | 0.361 | 14.091 (7.884 to 20.297) | <0.001 |
| Occlusion site: Carotid-T vs M1 | 198 | -2.495 (-11.500 to 6.509) | 0.588 | -0.681 (-3.566 to 2.203) | 0.644 | 1.805 (-1.073 to 4.684) | 0.220 |
| Occlusion site: M2 vs M1 | 198 | 0.142 (-7.767 to 7.483) | 0.971 | -1.147 (-3.589 to 1.296) | 0.359 | 2.182 (-0.255 to 4.620) | 0.081 |
| Right-sided occlusion | 198 | -2.181 (-8.304 to 3.942) | 0.486 | 0.770 (-1.186 to 2.727) | 0.441 | 0.391 (-1.659 to 2.441) | 0.709 |
| Prestroke mRS 3-5 vs 0-2 | 198 | 0.999 (-5.879 to 7.876) | 0.776 | -1.281 (-3.472 to 0.911) | 0.253 | 1.642 (-0.647 to 3.932) | 0.161 |
| Current smoker | 198 | 7.786 (-1.305 to 16.878) | 0.095 | 0.994 (-1.930 to 3.918) | 0.506 | 1.481 (-1.575 to 4.538) | 0.343 |
| Preinterventional CTA collateral score | 197 | -1.402 (-4.587 to 1.783) | 0.389 | 0.050 (-0.970 to 1.070) | 0.924 | -0.403 (-1.469 to 0.663) | 0.459 |
| Number of thrombectomy attempts | 197 | 0.047 (-1.214 to 1.308) | 0.942 | 0.536 (0.139 to 0.932) | 0.009 | 0.293 (-0.127 to 0.713) | 0.173 |

Supplementary Table 6. Exploratory regression analyses with NIHSS subscore item vigilance in a univariable and multivariable analysis (with age, sex, NIHSS, ASPECTS, occlusion site, prestroke mRS) with weight-adjusted dose rates as outcome

| **Exposures** | **N** | **Propofol (µg/kg/min)** | | **Remifentanil (ng/kg/min)** | | **Esketamine (µg/kg/min)** | |
| --- | --- | --- | --- | --- | --- | --- | --- |
|  |  | **Beta (95% CI)** | **p value** | **Beta (95% CI)** | **p value** | **Beta (95% CI)** | **p value** |
| NIHSS subscore vigilance, univariable analysis | 198 | -1.88 (-9.05 to 5.28) | 0.605 | -1.90 (-4.17 to 0.38) | 0.102 | -0.11 (-2.51 to 2.29) | 0.929 |
| NIHSS subscore vigilance, multivariable analysis | 198 | -1.71 (-9.59 to 6.16) | 0.668 | -2.18 (-4.67 to 0.30) | 0.085 | -0.13 (-2.58 to 2.31) | 0.914 |
